# A Delayed Foreign Body Reaction to a Hemostatic Agent Mimicking Neoplasm

**DOI:** 10.7759/cureus.95508

**Published:** 2025-10-27

**Authors:** Silvija Milanovic, Christina Sun, Tricia A Missall

**Affiliations:** 1 Department of Medicine, University of Florida College of Medicine, Gainesville, USA; 2 Department of Dermatology, University of Florida College of Medicine, Gainesville, USA

**Keywords:** dermato-pathology, foreign body reaction, hemostatic agent, hydrophilic polymer, potassium ferrate

## Abstract

Hydrophilic polymer-based hemostatic agents containing potassium ferrate are increasingly used in dermatologic surgery due to their rapid, coagulation-independent mechanism of action. While considered safe, delayed foreign body reactions to these agents are underrecognized and may mimic neoplastic recurrence, hypertrophic scarring, or cyst formation. We present the case of a 63-year-old man who developed a persistent papule at the site of a prior biopsy, clinically concerning for squamous cell carcinoma or residual cyst. Histopathologic evaluation revealed amorphous basophilic material with associated orange-red and yellow-brown granules, positive for iron on Prussian blue staining, surrounded by granulomatous and lymphohistiocytic inflammation. These findings were diagnostic of a foreign body reaction to hydrophilic polymer and potassium ferrate, likely introduced during the initial procedure. This case highlights the importance of recognizing the distinct histologic features of these reactions, especially when the procedural history is incomplete, to avoid misdiagnosis and unnecessary intervention. The prolonged latency, observed approximately one year after the initial procedure, emphasizes the need for continued vigilance by dermatopathologists and clinicians. As the use of topical hemostatic agents becomes more widespread, awareness of their potential for delayed foreign body reactions is essential for accurate diagnosis and appropriate management.

## Introduction

Hydrophilic polymer-based hemostatic agents, often combined with potassium ferrate, are used in dermatologic surgery to promote rapid clot formation in wounds healing by secondary intention [1-3]. These agents, marketed under names such as WoundSeal® and StatSeal®, function by absorbing plasma and oxidizing blood proteins to form a physical barrier, independent of the coagulation cascade [1-3]. They are particularly useful in patients with bleeding disorders or those on anticoagulants [1-3].

Although considered safe and widely available over-the-counter, foreign body reactions to hydrophilic polymer with potassium ferrate have been reported in a small but growing number of cases [2-5]. These reactions typically present weeks to months after application as persistent papules or nodules and may be mistaken for recurrent neoplasia, hypertrophic scarring, or cystic lesions [2,3]. Latency between exposure and presentation is variable, and delayed presentations (up to a year or more) may contribute to under-recognition of this association [6,7]. Histologically, the material appears as angulated, basophilic, non-polarizable fragments accompanied by yellow-brown and orange-red granules, often with granulomatous inflammation and multinucleated giant cells [2,3].

Correct identification of these features is essential to prevent misdiagnosis, particularly in re-excision specimens [3]. Distinguishing this material from other exogenous substances, such as ferric subsulfate (Monsel's solution), aluminum chloride, Gelfoam®, or dermal fillers, can be challenging [3-5,8-10]. Because these agents are commonly used and their histologic profiles are underrecognized, foreign body reactions may be underdiagnosed [2-5]. We report a case of histologically confirmed granulomatous inflammation due to hydrophilic polymer with potassium ferrate and review the key diagnostic features.

## Case presentation

A 63-year-old male presented with a solitary papule on the right upper back at the site of a prior punch biopsy for a presumed cyst approximately one year prior. On physical examination, there was a 0.9 cm tan papule with no associated symptoms. A gross image of the lesion was not included due to insufficient resolution for publication. The differential diagnosis included squamous cell carcinoma and recurrent or residual cyst. A shave biopsy was performed for histopathologic evaluation. 

**Figure 1:**
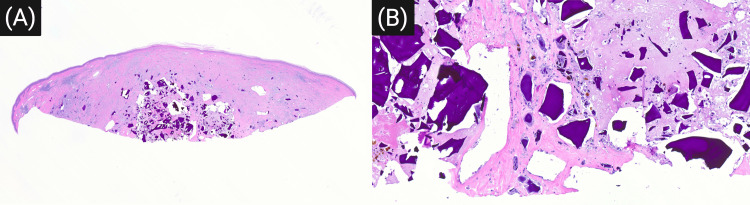
(A) Low-power H&E section showing dermal and subcutaneous granulomatous inflammation at the biopsy site (H&E, 1x magnification). (B) Higher magnification reveals amorphous, basophilic foreign material surrounded by multinucleated giant cells and lymphohistiocytic infiltrate (H&E, 10x magnification) H&E: Hematoxylin and Eosin

**Figure 2:**
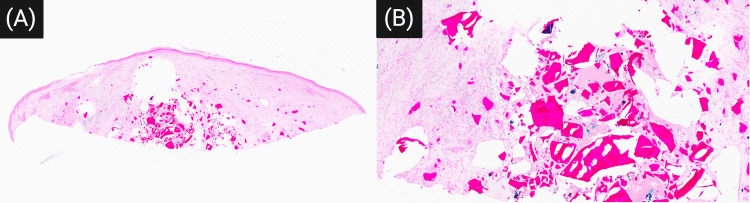
(A) Low-power Prussian blue stain highlighting scattered granular deposits within the dermis (Prussian blue, 1x magnification). (B) Higher-power view demonstrates iron-positive orange-brown granules associated with the foreign material, consistent with potassium ferrate (Prussian blue, 6x magnification)

Microscopic examination revealed dermal and subcutaneous granulomatous and lymphohistiocytic inflammation. There were numerous amorphous fragments of deep purple material (Figure 1A, 1B), surrounded by orange-red to brown granules that stained positive with Prussian blue (Figure 2A, 2B), indicating localized iron deposition associated with degradation of the hemostatic material. A narrow zone of overlying scar tissue was present, consistent with a prior biopsy site. The findings were diagnostic of a foreign body reaction due to hydrophilic polymer and potassium ferrate, likely introduced during the previous procedure.

## Discussion

Foreign body reactions to hydrophilic polymer-based hemostatic agents are becoming increasingly recognized in dermatopathology [2-5]. Although these agents are commonly used for their rapid and coagulation-independent hemostatic effects, the resultant tissue reactions can pose diagnostic challenges when they occur. In this case, a papule at a prior biopsy site was clinically suspicious for a new neoplasm, illustrating how such reactions can mimic malignancy and lead to unnecessary concern or intervention if incorrectly identified.

Histologically, the presence of angulated basophilic fragments, iron-positive granules, and granulomatous inflammation provided critical clues to the diagnosis (Figure 1A, 1B). These findings, consistent with previous reports, highlight the unique morphology of hydrophilic polymer with potassium ferrate, distinct from other common hemostatic agents or injected materials [2,3]. The iron-positive orange-brown granules observed in the dermis further support the presence of ferric salts derived from potassium ferrate (Figure 2A, 2B). The recognition of this pattern is particularly important given that procedural histories are often incomplete, especially when over-the-counter products are used or when hemostatic agents are not routinely documented in medical records, as in the case of our patient [2-5].

A notable feature in this case was the delayed presentation, approximately one year after the previous procedure. Prior literature reports variable latency ranging from weeks to months; however, longer delays may contribute to underrecognition of the association [2-7]. Dermatopathologists should consider hydrophilic polymer reactions in the differential diagnosis of persistent papules at prior procedural sites, especially when histologic features are suggestive but clinical history is inconclusive.

Differentiating this reaction from other exogenous materials remains essential. Monsel’s solution often appears as refractile brown pigment and may mimic spindle cell lesions, whereas aluminum chloride appears as fine basophilic cytoplasmic granules within histiocytes, which can resemble parasitized histiocytes [2,3,8-10]. Gelfoam® displays a distinct honeycomb-like structure and rarely elicits granulomatous inflammation [2-4,6]. In contrast, the deeply basophilic staining, angulated polymer fragments seen in this case are more characteristic of hydrophilic polymer, especially when accompanied by iron-positive debris [2,3]. All these can be seen in Table 1 below. 

**Table 1:** Histopathologic features of common foreign body materials PAS: Periodic acid-Schiff

Material/agent	Histologic appearance	Diagnostic features	Notes
Hydrophilic polymer (e.g., hemostatic agents)	Amorphous or angulated, deeply basophilic fragments; often surrounded by granulomatous inflammation and iron-positive debris	Prussian blue positive (iron deposition); non-refractile on polarization	Often associated with prior procedural site; delayed reaction possible
Monsel’s solution (ferric subsulfate)	Refractile brown pigment, may obscure cellular detail, and can mimic spindle cell proliferation	Positive for iron (Prussian blue); negative for melanin and hemosiderin stains	May mimic melanocytic or spindle cell lesions
Aluminum chloride	Fine basophilic cytoplasmic granules within histiocytes	Positive on aluminum stain; negative for iron	Can resemble parasitized histiocytes
Gelfoam® (gelatin sponge)	Honeycomb-like spaces or collapsed spongiform material and minimal inflammation	PAS positive; usually no iron deposition	Rarely elicits granulomatous reaction
Suture/gauze material	Refractile or birefringent fibers surrounded by foreign-body giant cells	Polarizable under polarized light	Common postoperative finding; usually non-pigmented
Talc/starch granules	Round, refractile, and sometimes Maltese-cross birefringence	Polarizable; negative for iron	Often an incidental contaminant

## Conclusions

This case contributes to the growing literature documenting the histopathologic spectrum of foreign body reactions to topical hemostatic agents. As the use of these agents becomes more widespread, increased awareness and familiarity with their tissue effects are essential to avoid misdiagnosis. Given the rarity of reported cases, continued reporting and characterization of such reactions will aid in refining diagnostic criteria and improving recognition among clinicians and pathologists.
